# The use of paediatric artemisinin combinations in sub-Saharan Africa: a snapshot questionnaire survey of health care personnel

**DOI:** 10.1186/1475-2875-10-365

**Published:** 2011-12-14

**Authors:** Selidji T Agnandji, Florian Kurth, Jose F Fernandes, Solange S Soulanoudjingar, Beatrice P Abossolo, Ghyslain Mombo-Ngoma, Arti Basra, Raquel González, Gondo Kizito, Pembe I Mayengue, Lorenz Auer-Hackenberg, Saadou Issifou, Bertrand Lell, Ayola A Adegnika, Michael Ramharter

**Affiliations:** 1 Medical Research Unit, Albert Schweitzer Hospital, Lambaréné, Gabon; 2 Institute for Tropical Medicine, University of Tübingen, Tübingen, Germany; 3 Department of Infectious Diseases and Pulmonary Medicine, Charité-Universitätsmedizin Berlin, Berlin, Germany; 4 Barcelona Centre for International Health Research (CRESIB), University of Barcelona, Barcelona, Spain; 5 Centro de Investigação em Saúde de Manhiça (CISM), Maputo, Mozambique; 6 Fondation Congolaise pour la Recherche Médicale/Faculté des Sciences de la Santé, Université Marien Ngouabi, Brazzaville, République du Congo; 7 Department of Medicine I, Division of Infectious Diseases and Tropical Medicine, Medical University of Vienna, Vienna, Austria; 8 Department of Parasitology, Leiden University Medical Center, Leiden, The Netherlands

**Keywords:** Artemisinin based combinations therapy, Paediatric drug formulation, Artemether, Lumefantrine, Amodiaquine, Dihydroartemisinin, Piperaquine, Mefloquine

## Abstract

**Background:**

Paediatric drug formulations for artemisinin combination therapy (P-ACT) have been developed over the past few years and have been shown to improve the therapeutic management of young children with uncomplicated falciparum malaria. This process was however not equally paralleled by a timely adoption of P-ACT in national and international treatment recommendations. National malaria programmes in sub-Saharan Africa have not yet widely embraced this new therapeutic tool. To which extent P-ACT is used in the field in sub-Saharan Africa is not known to date.

**Methods:**

This snapshot questionnaire survey aimed to provide an overview on the current routine practices for the availability and use of P-ACT as anti-malarial treatment for young children in sub-Saharan Africa. Health care personnel in seven countries in West-, Central, and East-Africa were invited to answer a structured questionnaire assessing use and availability of P-ACT.

**Results:**

A total of 71 respondents including doctors, nurses and pharmacy personnel responsible for the anti-malarial treatment of young children were interviewed. P-ACT was used by 83% (95% confidence interval: 73-90%; n = 59) as first-line treatment for young children. Use of 15 different P-ACT products was reported among which only two have received WHO prequalification status and approval by a stringent registration authority. Use of a specific P-ACT product was not linked to consumer prices or availability of supporting clinical trial data, but may depend more on the marketing capacity of the manufacturer. Major differences in frequency and dosing of anti-malarial regimens with identical anti-malarial compounds and the marketing of loose combinations were recorded.

**Conclusion:**

Paediatric ACT is widely used for the treatment of uncomplicated malaria in young children. However, the majority of P-ACT formulations in use do not meet highest international quality standards evoking concerns for patients' safety and the induction of drug resistance. Improving the quality of currently marketed P-ACT should constitute a public health priority besides their adoption into official treatment recommendations.

## Background

Several artemisinin combination therapies - the current standard of care for the treatment of uncomplicated falciparum malaria - have been recently developed in the form of paediatric formulations. These novel formulations combine several advantages compared to the conventional practice of crushing anti-malarial tablets for the treatment of young children including improved swallow ability, palatability, and more accurate dosing while maintaining high efficacy [1-9]. Importantly, the tolerability of drug administration was shown to be significantly improved in the treatment of children as evidenced by lower rates of drug induced vomiting and gastrointestinal disorders [10]. Based on this evidence, any paediatric artemisinin combination therapy (P-ACT) should have been taken up by national and international treatment guidelines as a novel tool to improve the management of uncomplicated malaria in young children - the most important target population suffering disproportionally from the burden of malaria.

Interestingly, major barriers to the adoption of P-ACT into national anti-malarial treatment policy in sub-Saharan Africa have been reported according to a recent survey [11,12]. Various reasons for this reluctance have been cited by national health authorities including a lack of effectiveness trials and unwillingness to diversify the product portfolio of national procurement agencies due to difficulties in supply management. Whereas P-ACT may not have been widely embraced by policy makers, little is known about the availability and use of anti-malarial treatments for young children in sub-Saharan Africa in the field [13]. In this snapshot survey, the current practice of anti-malarial treatment and the implementation of P-ACT as therapeutic option for young children were investigated in sentinel regions of sub-Saharan Africa.

## Methods

A questionnaire based survey investigating the use of anti-malarials for the treatment of young children was performed in seven sub-Saharan Africa. At least one country from West-, Central-, South, and East Africa where malaria is endemic and ACT constitutes the current first-line treatment for uncomplicated *Plasmodium falciparum *malaria was selected.

Doctors, nurses and pharmacists representing urban and rural areas as well as public and private sectors were invited by an investigator to answer a structured questionnaire. Eligible persons were informed about the nature and the general objectives of the study. Oral consent was obtained prior to the interview and information was recorded on paper case record forms. Interviews were conducted either face to face or on phone calls. Distinct questionnaires were developed for professionals working in clinical and pharmaceutical institutions in the country's official language. The survey was designed to cover the following topics: choice of first-line anti-malarial treatment in routine practice for young children (<5 years of age), current availability of anti-malarial drugs including trademarks and drug formulations, reasons for preference of anti-malarials, and attitudes towards official guidelines for the treatment of uncomplicated malaria. Pharmacists' questionnaires included questions about the current availability and actual cost of P-ACT for the patient and procurement procedures for anti-malarials. Data were coded and entered into a purpose built electronic database (Microsoft Excel 2010, Microsoft, WA). Descriptive univariate statistics were computed using a commercially available software package (JMP 6.0, SAS).

## Results

Seventy one health care professionals including 41 clinicians and 30 pharmacists participated in this survey from August 2010 until July 2011. Respondents were representing 23 municipalities, including nine urban, eight peri-urban and six rural localities in West- (Benin, Ivory Coast, Togo; n = 29), Central (Congo Republic, Gabon; n = 25), and South and East Africa (Mozambique, Uganda; n = 17) Clinicians participating in this survey were doctors and nurses responsible for the treatment of paediatric patients in the primary, district and tertiary public health care institutions (11, 13, and 17, respectively). Approximately half of the clinicians additionally work in private health care institutions. All pharmacists were employed in private pharmacies.

### Therapeutic options for the treatment of uncomplicated malaria in young children

ACT was reported as first-line anti-malarial treatment by all respondents (n = 71, 100%). ACT was cited as the second- or third-line treatment by 66 (93%) health professionals (not shown). Monotherapies including artesunate, amodiaquine and halofantrine as well as sulphadoxine-pyrimethamine combination were still available and were used as an alternative second-line treatment due to unavailability of ACT, mainly in rural areas.

Overall, 59 (83%, 95% confidence intervals: 73-90%) participants reported the use a P-ACT as the first-line drug for the treatment of uncomplicated *P. falciparum *malaria in young children (Tables 1 and 2). P-ACT formulations were still used as second line anti-malarial by 48 (73%) health professionals (Table 1).

**Table 1:** Anti-malarial drugs for the treatment of uncomplicated malaria in children: practices of health professionals of 7 sub-Saharan- African countries

Anti-malarial	First line treatment		Second line treatment		Third line treatment	
	
	Clinicians Pharmacists		Clinicians Pharmacists		Clinicians	Pharmacists
	N (%)	N (%)	N (%)	N (%)	N (%)	N (%)

***Paediatric ACT***						

AL	25 (61%)	24 (80%)	13 (34%)	12 (43%)	6 (25%)	11 (42%)

AS-AQ	6 (15%)	4 (13%)	11 (29%)	7 (25%)	1 (4%)	3 (12%)

AS-MQ						

DHA-PPQ			2 (5%)	3 (11%)		1 (4%)

**Total P-ACT**	**31 (76%)**	**28 (93%)**	**26 (68%)**	**22 (79%)**	**7 (29%)**	**15 (58%)**

***Tablets ACT***						

AL	7 (17%)	2 (7%)	1 (3%)	3 (11%)	2 (8%)	7 (26%)

AS-AQ	3 (7%)		3 (8%)		2 (8%)	1 (4%)

AS-MQ					2 (8%)	

DHA-PPQ			2 (5%)	1 (3%)	2 (8%)	

AS-S/P			1 (3%)	2 (7%)		

**Total T-ACT**	**10 (24%)**	**2 (7%)**	**7 (18%)**	**6 (21%)**	**8 (33%)**	**8 (30%)**

***Suppositories***			1 (3%)			1 (4%)

**Total ACT**	**41 (100%)**	**30 (100%)**	**34 (89%)**	**28 (100%)**	**15 (62%)**	**24 (92%)**

Paediatric monotherapy			4 (11%)		2 (8%)	1 (4%)

Tablet monotherapy					5 (20%)	1 (4%)

Total non-ACT monotherapy			**4 (11%)**		**7 (28%)**	**2 (8%)**

**Total respondents**	**41(100%)**	**30(100%)**	**38(100%)**	**28(100%)**	**24 (100)**	**26(100%**

*N *absolute number of respondents

%: percentage related to the absolute numbers

*ACT *Artemisinin Combination Therapy

*P-ACT *Paediatric Artemisinin Combination Therapy

*T-ACT *Tablet Artemisinin Combination Therapies (adult drug formulation)

*AL *artemether-lumefantrine; *AS *artesunate; *AQ *amodiaquine; *MQ *mefloquine; *DHA *dihydroartemisinin; *PPQ *piperaquine; *SP *sulfadoxine-pyrimethamine; *HF *halofantrine; *QNN *quinine

Clinicians: medical doctors and nurses

**Table 2:** Use of P-ACT in the treatment of uncomplicated falciparum malaria in young children: practices of health professionals of 7 sub-Saharan countries

	Clinicians N(%)	Pharmacists N(%)
Syrup, suspension	13(42%)	18(60%)

Dispersible tablet	16(52%)	8(27%)

Granules plus suspension	2(6%)	4(13%)

**Total**	31(100%)	30(100%)

*N*: absolute number

%: percentage

*ACT*: Artemisinin Combination Therapy

*P-ACT*: Paediatric Artemisinin Combination Therapy

*T-ACT*: Tablet Artemisinin Combination Therapy (adult like formulation)

Clinicians: medical doctors and nurses

Conventional tablet ACT was used by one fourth of clinicians in public health facilities participating in this survey as first line anti-malarials for the treatment of uncomplicated malaria in young children (n = 10, 24%), while all private pharmacists (n = 30, 100%) recommend or deliver under clinicians' prescription paediatric formulations as first-line treatment.

The availability and use of 15 different P-ACT products were reported by health professionals in this survey. The product portfolio included syrup, oral suspension, dispersible tablets, and granule formulations. A minimum of four formulations of P-ACT were listed by health professionals in Mozambique, and maximum of eight formulations of P-ACT in Gabon and Togo. Two formulations of P-ACT are not fixed-dose combinations and are marketed as loose combinations (amodiaquine syrup and artesunate granules). Among the 15 marketed combinations eight products are combinations of artemether and lumefantrine, representing 83% of the first-line P-ACT in this survey. Coartem^® ^dispersible, a WHO prequalified artemether-lumefantrine product, was given as first-line treatment by 24% of health professionals. Artesunate-amodiaquine was the second most used drug combination as first-line P-ACT (n = 10, 14%). Dihydroartemisinin-piperaquine and artesunate-mefloquine combinations were cited only as second- and third-line treatments (Table 1). Syrup (or powder for oral suspension) drug formulation was overall the preferred choice by health professionals followed by dispersible tablet drug formulations (Table 2).

Dosing recommendations for identical anti-malarial drug combinations varied markedly between different products. Artemether-lumenfantrine was found marketed as either a six-dose, three-day regimen, or as a once daily three-day regimen. Similarly dosing per kilogram bodyweight varied considerably between products of different manufacturers (Additional file 1).

### Regulatory status of P-ACT available on the market in sub-Saharan Africa

Out of the 13 fixed dose formulations of P-ACT reported in this survey, only Coartem^® ^dispersible tablets (artemether-lumefantrine) and Coarsucam^® ^dispersible tablets (artesunate-amodiaquine) have received WHO prequalification and in addition Coartem^® ^dispersible has received authorization by Swissmedic and the FDA.

### Sources of information for choices of anti-malarial treatment

Health care personnel were asked for the primary sources of information concerning the choice of anti-malarial treatment. Here, national guidelines were cited most frequently (n = 71, 100%). Medical representatives of pharmaceutical companies were the second most frequently cited source of information, particularly in urban and peri-urban regions (n = 57, 80%). Few professionals (n = 8, 11%) used external sources, including scientific publications or personal experience as informed basis for the decision for a first line anti-malarial.

### Cost of P-ACT

In all seven countries anti-malarials should be free of charge for children under five years of age, who are treated in public health facilities according to the interviewed professionals. In the private pharmacies interviewed in this survey, the cost of P-ACT ranged from 0.8 to 8.5 Euros. Dispersible tablets of artemether-lumefantrine and artesunate-amodiaquine, available as blisters, were the cheapest P-ACT (0.8-1.2 Euros for one treatment). Syrup, granules and oral suspension formulations costs ranged from 5 to 8.5 Euros.

## Discussion

This study was intended to provide an overview on the current availability and use of P-ACT in Africa. Today, it is known that official treatment recommendations and national policies are slow in incorporating this novel tool for the treatment and control of malaria. However, no information is available on the actual availability of P-ACT in sub-Saharan Africa, the current portfolio in this region, and most importantly the current use of P-ACT in the field.

This survey shows that P-ACT is used on a large-scale in sub-Saharan Africa, both by clinicians and by pharmacists. A surprisingly high number of participants reported the use of P-ACT as first-line treatment for young children. Importantly, even second- and third-line treatment constituted P-ACT in the majority of health care institutions. The artemether-lumefantrine combination was the most frequently prescribed P-ACT, accounting for 69% of first-line treatments in the study population. The preference of this ACT may be explained by multiple reasons. Firstly, artemether-lumefantrine constitutes the recommended first-line treatment in many African countries. Secondly, there is the highest number of generic artemether-lumefantrine combination products on the market totalling up to eight brandmarks of P-ACT. Finally, artemether-lumefantrine was the first internationally registered P-ACT on the market and may, therefore, have gained a competitive advantage and a larger market share. A high degree of market penetration and availability of P-ACT in sub-Saharan Africa has been shown in this survey. All (n = 30) pharmacies reported the availability of at least three different formulations of P-ACT. In Gabon, where this survey was initiated and had most interviews, a total of eight P-ACT brandmarks were available. Interestingly, P-ACT was similarly available in larger cities as in smaller communities in the interior provinces. These data show that - despite the fact that P-ACT is not procured on a large-scale by national procurement agencies - these drugs are effectively distributed and marketed by the private health care sector. Although, this study does not systematically investigate the availability of anti-malarials in public pharmacies, practices of health professionals working for public sector indicate that non-prequalified P-ACT are prescribed on a large scale in these institutions. In some regions including Gabon national health insurance covers and reimburses non-prequalified P-ACT prescribed to patients at public hospitals and health centres.

The current portfolio of P-ACT encompasses various kinds of child friendly drug formulations. Conventional syrup and solution drug formulations are available on a large-scale, as well as dispersible tablets, powder, and granules. Whereas the availability of all paediatric drug formulations is welcome, experts encourage the development and use of non-liquid paediatric drug formulations due to various considerations [14]. Stability of liquid drug formulations is often limited - particularly in hot climates - and production, distribution, and storage of liquids is by far more challenging and expensive due to higher volume and weight than dispersible tablet formulations [15-19].

Importantly, dosing regimens of anti-malarial combination therapy varied between manufacturers. Whereas artemether-lumefantrine tablets showed markedly diminished efficacy in dosing regimens of less than six intakes over three days, liquid artemether-lumefantrine formulations are marketed as a once daily three-day treatment (Additional file 1). Since there is lack of data from high quality clinical trials for many of artemether-lumefantrine products, differences in dosing strengths and frequency raise important concerns. Importantly, two forms of P-ACT are marketed as loose drug combinations. This practice is contrary to current recommendations, since loose drug combinations are associated with reduced patient adherence and erroneous dosing. Moreover, artesunate-monotherapy is included in these products, a practice that is banned by the World Health Organization due to considerations regarding the development of artemisinin drug resistance. Given the high number of alternative forms of P-ACT, the marketing of these loose combinations seems not warranted.

Taste-masking is another important feature of P-ACT intending to conceal the intensely bitter taste of most anti-malarial agents. This may be achieved by a drug formulation produced to avoid any taste of the product or by masking and adding a specific well appreciated flavour. The reported product portfolio consists of a majority of taste-masked products. Some exceptions, including the WHO prequalified ASAQ (Sanofi-Aventis and DNDi), are not taste-masked and exhibit, therefore, a bitter taste. To our knowledge no data on the comparative acceptability of various drug formulations in the treatment of young children are available so far. It is unclear whether specific forms of paediatric drug formulations or whether taste-masking may improve the acceptability of P-ACT and this should become an area of active research in the future.

In this survey, it was also intended to elucidate reasons for the choice of specific anti-malarials by health care providers. Reassuringly, national treatment recommendations were cited as primary source in all regions. However, this refers more to recommendations for combinations of anti-malarial substances rather than for specific drug formulations or specific products. This situation may explain why P-ACT, although not specifically recommended by most national treatment guidelines, is used on a large scale, and also why some anti-malarial brands are used on a large scale, even if not supported by clinical trial data. Importantly, medical representatives rank second as a source of information and are by far more important than external sources, including scientific publications and internet resources. This situation seems problematic since balanced information including quality and cost of P-ACT may not be provided and artful marketing may be the decisive advantage for or against the choice of an anti-malarial.

This survey is the first to address the issue of real world use and availability of P-ACT in several sub-Saharan Africa countries and was conducted to provide an overview on the current situation both for researchers as well as for policy makers. Despite a surprisingly high coverage and diversity of the market with P-ACT, inherent limitations of this study need to be acknowledged. This survey was not designed to provide representative data for all of Africa, since more resources would have been necessary to perform random sampling and high coverage in urban and remote regions of all major African regions. Random sampling was not performed and selection of participants was influenced by their accessibility. It is, therefore, understood that this survey may not accurately reflect subtle differences in treatment practices, at a regional and sub-regional level, nor at public versus private health sectors. However, this snapshot assessment of routine practices of health care providers in sub-Saharan Africa has provided important information about the current role of P-ACT in the treatment of African children. Further data from surveys with larger sample size are needed to strengthen the answers gathered in this snapshot survey. Since no systematic information has been available so far, these data may serve as valuable evidence for researchers and policy makers alike.

## Conclusions

In summary, P-ACT is used on a large scale in sub-Saharan Africa and availability of various brands is surprisingly high. However, quality issues of distinct formulations of P-ACT are underappreciated by health care providers and resources are often diverted to the purchase and use of products without sufficient clinical evidence. Objective guidance to support the choice of the selected P-ACT is necessary for both national authorities and health care personnel.

## Competing interests

The authors declare that they have no competing interests.

## Authors' contributions

AST and MR have designed the survey and drafted the manuscript. SSS, JFF, BPA, KF, IMP, SB, SI, BL, AAA, MR have performed the interviews and participated in manuscript preparation. All authors have reviewed and approved the final version of the manuscript.

## Supplementary Material

Additinal file 1 **P-ACT listed by health professionals in 7 sub-Saharan countries: trademarks, formulations, regimen, daily dosage and regulatory status**. Click here for file
