# Supplementary material for: The use of paediatric artemisinin combinations in sub-Saharan Africa: a snapshot questionnaire survey of health care personnel
Source: Malar J. 2011 Dec 14;10:365. doi: 10.1186/1475-2875-10-365 (PMC3281189; doi:10.1186/1475-2875-10-365)
Supplement: Additinal file 1 — P-ACT listed by health professionals in 7 sub-Saharan countries: trademarks, formulations, regimen, daily dosage and regulatory status. [file 1475-2875-10-365-S1.DOC]

**Additional File 1.**

**P-ACT listed by health professionals in 7 sub-Saharan countries: trademarks, formulations, regimen, daily dosage and regulatory status.**

| Products | Manufacturer | Formulation | Regimen | Recommended daily dosage | Regulatory status |
| --- | --- | --- | --- | --- | --- |
| **Artemether-Lumefantrine** |  |  |  |  |  |
| Coartem**®** | Novartis Pharma, Switzerland | Dispersible tablet | Twice daily | A: 40mg L:240mg | WHO prequalification,  FDA approval |
| Co-Artesiane**®** | Dafra Pharma International, Belgium | Syrup, powder/Suspension | Once daily | A: 21-54mg L:126-324mg | Approval at national level |
| Lufanter**®** | IMEX Group, Switzerland and India | Powder/Suspension | Once daily | A: 21-54mg L:126-324mg | Approval at national level |
| Cofantrine**®** | IMEX group, Switzerland and India | Powder/Suspension | Once daily | A: 21-54mg L:126-324mg | Approval at national level |
| Bimalaril**®** | Medical pharmaceutique, France | Powder/Suspension | Once daily | A: 40mg L:240mg | Approval at national level |
| Lonart**®** | Bliss GVS Pharma Limited, India | Powder/Suspension | Once daily | A: 21-54mg L:126-324mg | Approval at national level |
| Lum-Art plus**®** | Unknown | Powder/Suspension | Once daily | A: 21-74mg L:126-324mg | Approval at national level |
| Artefan**®** | Ajanta pharma, India | Powder/Suspension | Once daily | A: 21-74mg L:126-324mg | Approval at national level |
| **Artesunate-Amodiquine** |  |  |  |  |  |
| Coarsucam**®** | Sanofi-Aventis, France | Dispersible tablets | Once daily | AS: 50mg AQ:135mg | WHO prequalification |
| Camoquin-plus**®** (not FDC) | Pfizer, USA | Syrup plus granules | Once daily | AS: 50mg AQ: 150mg | Approval at National level |
| Artepal**®** (Not FDC) | Olea, Ivory coast and France | Syrup plus granules | Once daily | AS: 50 mg AQ: 150 mg | Approval at National level |
| Artediam**®** | Odypharm, UK | Powder/ suspension | Once daily | AS: 50mg AQ: 150mg | Approval at national level |
| **Dihydroartemisinin-piperaquin** |  |  |  |  |  |
| Malacur**®** | Salvat pharmaceutical, Spain | Syrup | Twice Day 1; Once D2& D3 | DHA: 45 / 22.5mg  PQ: 360/180mg | Approval at national level |
| P-Alaxin**®** | Bliss GVS Pharma Limited, India | Powder/Suspension | Twice Day 1; Once D2& D3 | DHA: 40 / 20mg  PQ: 320/160mg | Approval at national level |
| **Artesunate -Mefloquine** |  |  |  |  |  |
| Artequin**®** | Mepha, Switzerland | Granules | Once daily | AS: 50 mg MQ: 125mg | Approved at national level |

AL (Artemeter-Lumefantrine): dosage for children 5 kg to less than 15 kg

ASAQ (Artesunate-Amodiaquine): dosage for children between 10 and 20 kg

DHA-PQ (Dihydroartemisinin-Piperaquine): dosage for children between 1-7 years. Dosages at day 1 and subsequent days

ASMQ (Artesunate-Mefloquine): dosage for children between 10 and 20 kg

WHO (World Health Organization)

FDA (Food and Drug Administration)

Not FDC : Not-Fixed Dose Combination = loose formulations of the two compounds
